# Utility-based Analysis of Statistical Approaches and Deep Learning Models for Synthetic Data Generation With Focus on Correlation Structures: Algorithm Development and Validation

**DOI:** 10.2196/65729

**Published:** 2025-03-20

**Authors:** Marko Miletic, Murat Sariyar

**Affiliations:** 1 Institute for Optimisation and Data Analysis (IODA) Bern University of Applied Sciences Biel Switzerland

**Keywords:** synthetic data generation, medical data synthesis, random forests, simulation study, deep learning, propensity score mean-squared error

## Abstract

**Background:**

Recent advancements in Generative Adversarial Networks and large language models (LLMs) have significantly advanced the synthesis and augmentation of medical data. These and other deep learning–based methods offer promising potential for generating high-quality, realistic datasets crucial for improving machine learning applications in health care, particularly in contexts where data privacy and availability are limiting factors. However, challenges remain in accurately capturing the complex associations inherent in medical datasets.

**Objective:**

This study evaluates the effectiveness of various Synthetic Data Generation (SDG) methods in replicating the correlation structures inherent in real medical datasets. In addition, it examines their performance in downstream tasks using Random Forests (RFs) as the benchmark model. To provide a comprehensive analysis, alternative models such as eXtreme Gradient Boosting and Gated Additive Tree Ensembles are also considered. We compare the following SDG approaches: Synthetic Populations in R (synthpop), copula, copulagan, Conditional Tabular Generative Adversarial Network (ctgan), tabular variational autoencoder (tvae), and tabula for LLMs.

**Methods:**

We evaluated synthetic data generation methods using both real-world and simulated datasets. Simulated data consist of 10 Gaussian variables and one binary target variable with varying correlation structures, generated via Cholesky decomposition. Real-world datasets include the body performance dataset with 13,393 samples for fitness classification, the Wisconsin Breast Cancer dataset with 569 samples for tumor diagnosis, and the diabetes dataset with 768 samples for diabetes prediction. Data quality is evaluated by comparing correlation matrices, the propensity score mean-squared error (pMSE) for general utility, and *F*_1_-scores for downstream tasks as a specific utility metric, using training on synthetic data and testing on real data.

**Results:**

Our simulation study, supplemented with real-world data analyses, shows that the statistical methods copula and synthpop consistently outperform deep learning approaches across various sample sizes and correlation complexities, with synthpop being the most effective. Deep learning methods, including large LLMs, show mixed performance, particularly with smaller datasets or limited training epochs. LLMs often struggle to replicate numerical dependencies effectively. In contrast, methods like tvae with 10,000 epochs perform comparably well. On the body performance dataset, copulagan achieves the best performance in terms of pMSE. The results also highlight that model utility depends more on the relative correlations between features and the target variable than on the absolute magnitude of correlation matrix differences.

**Conclusions:**

Statistical methods, particularly synthpop, demonstrate superior robustness and utility preservation for synthetic tabular data compared with deep learning approaches. Copula methods show potential but face limitations with integer variables. Deep Learning methods underperform in this context. Overall, these findings underscore the dominance of statistical methods for synthetic data generation for tabular data, while highlighting the niche potential of deep learning approaches for highly complex datasets, provided adequate resources and tuning.

## Introduction

In recent years, Generative Adversarial Networks (GANs) and large language models (LLMs) have revolutionized the synthesis and augmentation of medical data [1-3]. These technologies have introduced methods for creating high-quality, realistic datasets, which are essential for advancing machine learning (ML) applications in the health care sector [4-6]. The ability to synthesize realistic medical data is particularly valuable in contexts where data privacy and availability are major concerns [7]. Medical data is often subject to strict regulations due to privacy laws and ethical considerations, which can limit the availability of comprehensive datasets for research and development. By using GANs and LLMs to generate synthetic data, researchers and practitioners can overcome these limitations, creating datasets that preserve the statistical properties and correlations of the original data while ensuring that individual patient identities remain protected.

However, despite the promising capabilities of GANs and LLMs, several challenges persist in leveraging these technologies effectively for medical data synthesis [8-11]. A key challenge is the ability of these models to accurately capture and replicate the intricate relationships within medical datasets. Medical data often exhibits complex interdependencies between features, such as the relationship among symptoms, diagnostic indicators, and treatment outcomes. Inaccurate representation of these correlation structures can result in synthetic data that fails to mimic the true variability and relationships found in real-world medical data [12]. The use of synthetic medical data also raises ethical concerns, particularly regarding the potential perpetuation or, in some cases, even amplification of biases inherent in the original datasets [13]. For instance, GANs tend to prioritize matching overall data distribution rather than subgroup-level details. Such representation issues can translate into new or stronger associations between sensitive attributes such as race and medical conditions [14]. If high data quality is promised based on such data because a particular metric performs well, ML methods may establish incorrect associations accordingly.

Focusing on pairwise correlation structures in medical data synthesis, despite their limitations in complex data environments, remains crucial for several reasons: (1) correlation analysis identifies primary dependencies as a starting point for understanding how variables interact; (2) if a ML model recognizes that certain variables are typically correlated, it can better simulate realistic scenarios, leading to more accurate predictions and insights; and (3) pairwise correlation structures provide a baseline for validating and comparing synthetic data. Even though they might not capture all forms of dependence, comparing correlations in synthetic data with those in real-world data can help assess the fidelity and quality of the generated datasets.

There have been several approaches addressing correlations in the context of Synthetic Data Generation (SDG), particularly for relational data [15]. Most methodological studies aim to capture correlation structures by extending existing techniques. For example, Vu et al [16] explored how to make the loss function of GANs correlation-aware but found no significant benefit. In contrast, Patel et al [17] demonstrated that incorporating a Correlational Neural Network can improve a GAN’s ability to capture correlations, slightly outperforming the MedGAN model. Torfi and Fox developed realistic synthetic health care records by leveraging Convolutional Neural Networks to capture correlations between medical features, achieving comparable performance to real data in ML tasks while maintaining privacy and statistical fidelity [18]. Rajabi and Garibay [19] showed that effective consideration of correlations can enhance fairness in synthetic data. These works are noteworthy because the primary goal of advanced SDG methods is to capture the full dependency structure.

Despite the substantial body of work on validation and benchmarking in SDG, there is a notable gap in studies specifically assessing how the correlation structure of real data influences the effectiveness of SDG methods in replicating such relationships. Understanding whether faithfully reproducing correlation structures is critical for achieving high-quality results in downstream tasks remains an open question. This issue is particularly relevant given the increasing reliance on SDG methods across various domains. Simulation studies are well-suited to address these questions, as they enable controlled analysis of specific factors affecting model performance [20]. For instance, Strobl et al [21] demonstrated through simulations that Random Forest (RF) models tend to produce biased variable selection when predictors differ in scale or category count.

The aim of this study is to address the research gap by developing a simulation design and validating the results on 3 real-world medical datasets. We evaluate how effectively SDG methods can replicate the correlation structure of the original data and perform a classification task using RF. To provide a comprehensive analysis, alternative models such as eXtreme Gradient Boosting [22] and Gated Additive Tree Ensembles [23] are also considered. In addition, for one notable case, we assess whether the relevant variables are selected based on variable importance measures, as correlation matrix distances are often calculated in practice without addressing their impact. For this analysis, we use the following SDG approaches: Synthetic Populations in R (synthpop) [24], copula [25], copulagan [26], Conditional Tabular Generative Adversarial Network (ctgan) [27], Tabular Variational Autoencoder (tvae) [27], and tabula for LLMs [28,29], the latter of which per default uses DistilGPT-2 (distilled Generative Pretrained Transformer -2), a streamlined version of the english-language model GPT-2. The corresponding assessment will help practitioners in guiding their choice of SDG methods.

## Methods

### Overview

**Figure 1:**
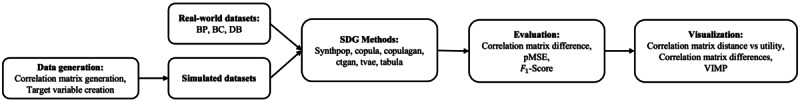
Overview of the methodology workflow. BC: Breast Cancer Dataset; BP: Body Performance Dataset; ctgan: Conditional Tabular Generative Adversarial Network; DB: Diabetes Dataset; pMSE: Propensity Score Mean-Squared Error; SDG: synthetic data generation; tvae: Tabular Variational Autoencoder; VIMP: variable importance.

The schematic diagram in Figure 1 outlines the key steps in the methodology used in this study. The process begins with data generation, where simulated datasets were created using correlation matrix construction and target variable creation. Besides that, we selected 3 real-world datasets (Body Performance [BP], Breast Cancer [BC], and Diabetes [DB]). All datasets are then used to generate and evaluate various SDG methods.

### Datasets

#### Real-World Datasets

**Figure 2:**
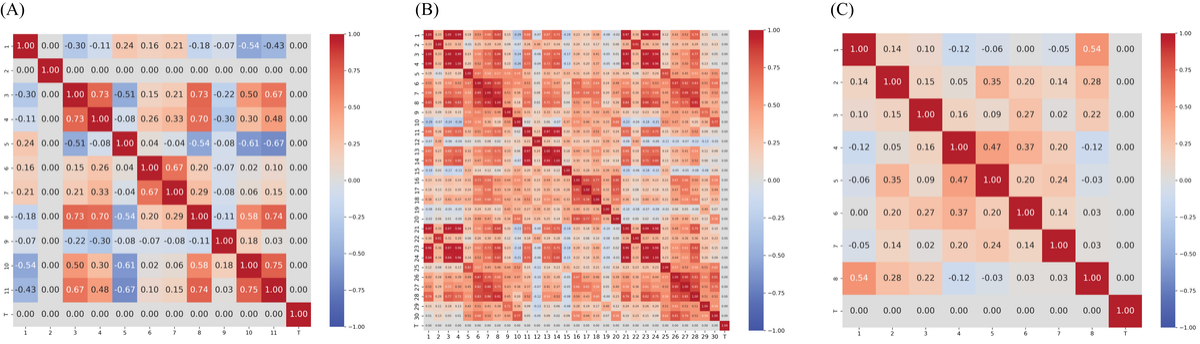
Correlation matrix for 3 real-world datasets: (A) BP: Body Performance Dataset, (B) BC: Breast Cancer Dataset, and (C) DB: Diabetes Dataset.

We selected 3 medical datasets from Kaggle – Body Performance (BP), Breast Cancer (BC), and Diabetes (DB) – that are commonly used in predictive modeling and data analysis tasks. All 3 datasets involve classification problems. The correlation matrices of these datasets are provided in Figure 2.

The BP dataset provides comprehensive data on physical fitness and body measurements, encompassing variables such as height, weight, age, gender, body fat percentage, and details of physical activity and fitness routines. It includes 13,393 samples with 11 numerical features and a categorical target variable that classifies individuals into four fitness categories: excellent, good, average, and poor. Among the features, age and sit-up count are recorded as integers.

The BC dataset comprises 569 entries, each with 30 numerical features extracted from digitized images of fine needle aspirates of breast masses. These features, representing the mean, standard error, and maximum value, quantify geometric and textural properties of cell nuclei, including radius, texture, perimeter, area, smoothness, compactness, concavity, concave points, symmetry, and fractal dimension. The dataset supports tumor classification as malignant or benign based on the nuclei features.

The DB dataset is tailored for predicting diabetes based on diagnostic measurements. It comprises 768 records of Pima Indian women aged 21 and older, with variables including the number of pregnancies, glucose levels, blood pressure, skin thickness, insulin levels, BMI, a diabetes pedigree function, age, and a binary diabetes outcome. All variables are numerical, representing physiological and diagnostic metrics critical to diabetes prediction.

#### Simulated Datasets

In our simulation study, we first generate 10 Gaussian-distributed features and then impose distinct correlation structures using the Cholesky decomposition method [30]. A binary target variable is subsequently constructed based on 4 selected features. The process of defining the target variable is repeated across 3 different correlation structures, with the simulation executed at 3 distinct sample sizes (500, 5000, and 10,000). The use of varying sample sizes allows us to examine the effect of data volume on the robustness and stability of the correlation structures and the resulting relationships between features and the target variable.

To introduce correlations, we construct 3 types of correlation matrices based on 3 different exponential decay rates, corresponding to varying strengths and patterns of correlation: 0.1 for strong positive correlations, 0.3 for weaker positive correlations, and 0.25 for alternating correlations (positive and negative). The correlation between variables is defined using equation (1) for the 0.1 and 0.3 decay rates, where the exponential decay ensures that correlations decrease as the index distance increases:



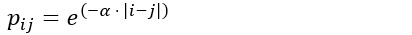



Here, α represents the decay rate, controlling the speed at which correlations diminish as the distance |*i* – *j*| between indices grows. Smaller values of (eg, 0.1) result in slower decay and stronger correlations over larger distances, while larger values (eg, 0.3) lead to faster decay and weaker correlations.



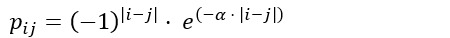



**Figure 3:**
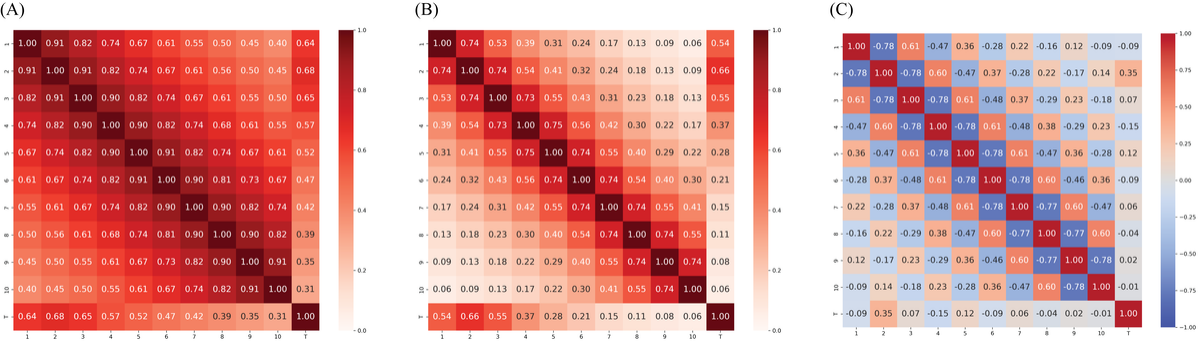
Correlation matrices used in the simulation study: (A) positive exponential decay rate of 0.1, (B) positive exponential decay rate of 0.3, and (C) alternating positive and negative exponential decay rate of 0.25.

For the 0.25 alternating correlation, equation (2) is used, incorporating alternating signs to produce correlations that switch between positive and negative values with increasing index distance. In this case, *α* = .25 determines the rate of decay, while the alternating factor (–1)^|i – j|^ introduces the sign changes in the correlations. The resulting correlation matrix, which must fulfill the condition of symmetric positive semidefiniteness, is then decomposed via Cholesky decomposition, allowing us to transform independent normal variables into correlated ones as defined by the specified structure. Examples of such generated correlation matrices are shown in Figure 3.

The correlation between different types of variables is calculated through a structured process that accommodates binary, continuous, and mixed data types. For each pair of variables, the appropriate correlation metric is selected based on their data types. If at least one variable is binary, the Point-Biserial correlation coefficient is used [31]. The data with the correlated variables is then used to construct a binary target variable, which is defined as a linear combination of the first 4 features from the 10 generated variables, as shown in equation (3):



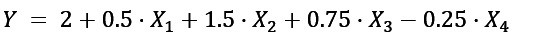



The remaining 6 variables *(X_5_, … , X_10_)* do not contribute to *Y* and effectively act as noise variables in the dataset. These noise variables introduce additional complexity by creating scenarios where irrelevant features must be disentangled. This setup mimics real-world scenarios where datasets often contain features that are unrelated or weakly related to the target variable. *Y* is then used to define thresholds based on its median, with a range of SD 10% around the median. Values exceeding the upper threshold are assigned the binary label 1, while those below the lower threshold are assigned 0. For values within the threshold range, binary labels are assigned randomly. It should be noted that while the features *X_1_, X_2_, X_3_, X_4_* remain continuous, the binary target variable is derived through this thresholding approach applied to the linear combination defined in equation (3).

The complexity in these simulated datasets arises from structured correlation patterns, where the strength, direction, and interplay of correlations among features significantly affect their relationships with the target variable. This correlational complexity can be understood at three levels:

1. Feature-target correlation: Variability in how individual features relate to the target, ranging from strong to very weak associations.
2. Feature-feature correlation: Associations among features that introduce complicate the disentanglement of their individual contributions to the target.
3. Global correlation structures: The overall arrangement of feature-target and feature-feature correlations, encompassing uniform (eg, consistent signs) or mixed configurations (eg, alternating signs).

Based on these levels, the datasets can be categorized into three complexity groups:

- Low complexity: Features exhibit rather strong relationships with the target, minimal or no correlations among features, and homogeneous global correlation.
- Moderate complexity: Feature-target relationships vary, ranging from strong to weak, with moderate feature-feature correlations, and consistent correlation signs.
- High complexity: Feature-target relationships are rather weak, with moderate feature-feature correlations, and alternating correlation signs (Figure 3C).

As complexity increases, the challenges in data analysis and modeling grow substantially. The correlation matrices of both simulated and real data reveal that BP most closely aligns with the 0.25 case (high complexity), BC with the 0.1 case (low complexity), and DB with the 0.3 case (low complexity).

### Synthetic Data Generation Methods

We use a range of SDG methods to explore diverse approaches to data synthesis. Statistical methods include synthpop, a widely used statistical model that generates synthetic data by fitting individual features and their conditional distributions based on the observed data structure. Synthpop is particularly well-suited for datasets with both continuous and categorical variables, as it applies models such as classification and regression trees that account for different data types. Another statistical method, copula, uses copula functions to model dependencies among variables, allowing for the generation of multivariate synthetic data by combining marginal distributions with a dependency structure. While copula-based methods are primarily designed for continuous variables, extensions or preprocessing techniques can be used to encode and incorporate categorical variables, such as one-hot encoding or ordinal transformations.

For more advanced generative approaches, we use copulagan, ctgan, and tvae, which are deep learning–based models designed to handle complex data synthesis tasks. Copulagan combines the dependency modeling capabilities of copulas with GANs. It learns the marginal distributions of real data columns and applies ctgan to model normalized data, improving the synthesis of mixed data types. Ctgan uses conditional GANs to address challenges in imbalanced and categorical data. It incorporates techniques like mode-specific normalization to handle high-cardinality categories, enabling precise modeling. Tvae captures complex, nonlinear relationships in tabular data by learning latent representations and generating high-quality synthetic data. In addition, we used the Tabula [29] LLM, which leverages LLMs such as a distilled Generative Pretrained Transformer-2 model, and encodes tabular data into natural language-style representations. This framework allows flexible data generation, incorporating domain-specific contexts and enabling synthesis from textual prompts. While not all models used qualify as LLMs (parameter sizes ≥1 billion), we used the term for simplicity.

For the implementation of copula, copulagan, ctgan, and tvae we used the Synthetic Data Vault library (SDV [32]). SDV (Andrew Montanez et al) integrates various methods into a unified framework, facilitating seamless experimentation and evaluation. Although adaptations of synthpop for Python (Sam Maurer et al) exist, we used the native R [24] environment, as it provides the most stable and comprehensive implementation.

### Utility and Correlation Matrix Distance Measures

To evaluate the quality of the synthetic data, we use 3 key metrics. First, training on synthetic data and testing their performance on original data, using the *F*_1_-score as a measure. The *F*_1_-score is calculated using a classification probability cutoff of 0.5. This approach is often referred to as train-synthetic-test-real. The evaluation differs depending on whether the data is derived from real-world datasets or simulated datasets. For real-world datasets, the original data is split into training and testing sets with an 80/20 split. The 80% training split is used to train the SDG methods, and an equivalent amount of synthetic data (corresponding to the 80% training size) is generated. The quality of this synthetic data is then evaluated by testing it against the original 20% testing split from the real-world dataset. For simulated datasets, 100% of the “real” simulated data is used to train the SDG methods. To evaluate the quality of the synthetic data, a separate test set consisting of 100% newly generated synthetic data was created. The performance is then assessed by testing the synthetic simulated data against the “real” simulated data containing the full 100% of the samples. The *F*_1_-score resulting from training on the original data is represented as a dashed line in the visualizations.

Second, we compute the squared differences between the correlation matrices of the original and synthetic datasets. This metric quantifies the extent to which the synthetic data replicates the pairwise correlations present in the original data. Finally, we use the propensity score mean-squared error (pMSE), which is a metric used to evaluate the utility of synthetic data by measuring the distinguishability between real and synthetic datasets. It is defined as:



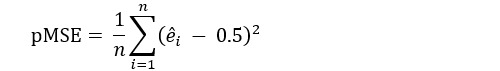



Where *ê_i_* represents the estimated propensity score for the *i-*th observation, which measures the probability of a sample being synthetic rather than real. The goal of synthetic data generation is to create data so realistic that the model cannot easily distinguish between synthetic and real samples. Therefore, lower pMSE values indicate better performance, as they imply a higher degree of similarity between the real and synthetic datasets. A pMSE value close to 0.25 (the maximum achievable value when synthetic and real datasets are highly distinguishable) suggests bad synthetic data generation [33]. Normalizing this metric by dividing it with 0.25 leads to values between 0 (indistinguishable) and 1 (highly distinguishable).

### Variable Importance Measures

Python machine learning libraries, for example, sklearn, typically provide various methods to calculate variable importance (VIMP). The main two approaches are (1) Gini importance and (2) permutation importance [34]. Gini importance measures the reduction in Gini impurity when a feature is used to split a node. The feature’s importance is quantified by the total decrease in impurity across all trees. Features that contribute more to impurity reduction are considered more important, although this method can be biased toward features with more categories or higher cardinality.

Alternatively, permutation importance evaluates a feature’s significance by measuring the drop in model performance, typically accuracy, when the feature’s values are randomly shuffled. The importance score is derived from the change in performance on out-of-bag samples before and after shuffling. A larger decrease in accuracy indicates greater importance. This method is more robust, accounting for feature interactions and reducing biases, but is computationally more demanding.

Using both Gini importance and permutation importance provides complementary insights: Gini impurity reflects a feature’s contribution to better splits within trees, while permutation-based importance directly measures a feature’s impact on overall prediction accuracy. Combining both methods offers a more balanced assessment of feature relevance.

### Evaluation Design

We conduct 10 sampling iterations for each combination of SDG methods. For deep learning approaches, we evaluate training epoch sizes of 300, 1000, and 10,000 on both simulated and real datasets. For LLMs, we limit the epoch sizes to 300 and 1000 due to significantly higher resource demands and previous findings indicating no performance improvement with larger epoch counts [35]. The batch size is fixed at 500 for the deep learning SDV methods and 64 for LLMs. Specifically, we compute the mean *F*_1_-score and correlation matrix differences across the 10 samples for each SDG method and epoch size. For the most notable results, we visualize the correlation matrix differences and calculate the VIMP scores for the best and worst-performing methods.

## Results

We will first present the results for the simulated data, followed by those for the real data. Since the results from eXtreme Gradient Boosting and Gated Additive Tree Ensembles are nearly identical to those from Random Forest and provide no additional insights, we have omitted them here (Multimedia Appendix 1). Although we anticipated this outcome, we sought to empirically validate it. The analysis will then continue with an examination of the VIMP scores and visualization of the correlation distances for the most notable case, which simulated data consisting of 10,000 samples with an alternating decay parameter of 0.25. This scenario is chosen because it illustrates a case where, despite a large sample size, there is a considerable performance gap between the best- and worst-performing methods.

### Correlation Distance and Utility Comparison

#### Simulated Data

**Figure 4:**
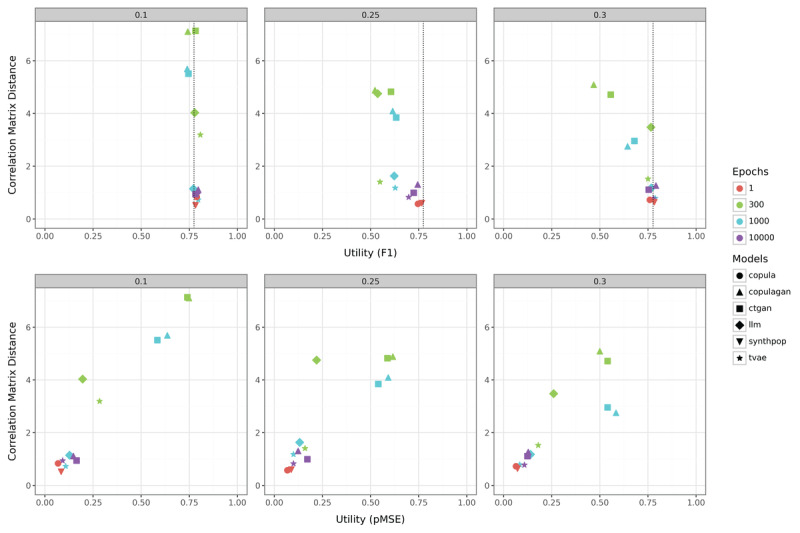
Comparison of the correlation matrix distance and utility metrics (F_1_-score in the top row; pMSE in the bottom row) for the simulated dataset with a sample size of 500. ctgan: Conditional Tabular Generative Adversarial Network; LLM: large language model; pMSE: Propensity Score Mean-Squared Error; synthpop: Synthetic Populations in R; tvae: Tabular Variational Autoencoder.

Figure 4 presents the results of our methods on the smallest simulated dataset with 500 samples. For the case of strong positive correlations (0.1), there is virtually no difference in utility between generated and original simulated data. In other words, most models cluster tightly around a RF utility of approximately 0.75. Some models (eg, ctgan and copulagan at 300 and 1000 epochs) have higher correlation matrix distances, indicating weaker preservation of correlation structures. Deep learning models trained with more epochs (eg, 1000 or 10,000, indicated by blue and purple) perform better in terms of correlation matrix distances compared to models with 300 epochs. In terms of utility, epoch sizes do not have a significant effect in this scenario because the data complexity seems not high enough to require prolonged training. The observation that utility remains unaffected by high correlation matrix distances highlights that a poor approximation of the correlation structure is problematic only under specific conditions.

In the scenario with moderate positive correlations (0.3), the higher correlation distance of ctgan and copulagan at low epoch counts now also negatively affects the RF utility, despite the correlation matrix distance being lower than in the case of 0.1. The pMSE values are overall lower, suggesting that the increased complexity primarily affects the RF utility. Models trained with 10,000 epochs again demonstrate improved performance, characterized by lower correlation matrix distances and enhanced RF utility, although the pMSE values are higher. The relationship between pMSE values, correlation matrix differences, and RF utility is demonstrated by comparing LLM with 300 epochs and ctgan with 1000 epochs: while LLM exhibits a higher correlation matrix difference, its superior utility results in a significantly lower pMSE value overall. As observed in the 0.1 case, tvae and LLM with high training epochs again rank among the top-performing methods in this scenario, with copula and synthpop achieving the highest performance. The same necessity for extended training epochs as in the 0.1 case suggests that deep learning models likely struggle due to insufficient training data.

In the most complex scenario (0.25), the performance of each SDG method in RF utility is worse than with the original data. This is particularly evident as the tvae and LLM models deviate more significantly from the baseline even with 10,000 epochs. However, these differences have minimal impact on the pMSE values, where copula and synthpop consistently emerge again as the best-performing methods. The high complexity of this simulated dataset primarily manifests as reduced RF utility rather than increased pMSE. However, the differences compared with the 0.3 scenario are not substantial. Notably, well-performing methods show remarkable robustness, while deep learning approaches with fewer epochs, typically recommended as default settings for practical applications, perform surprisingly poorly by comparison.

**Figure 5:**
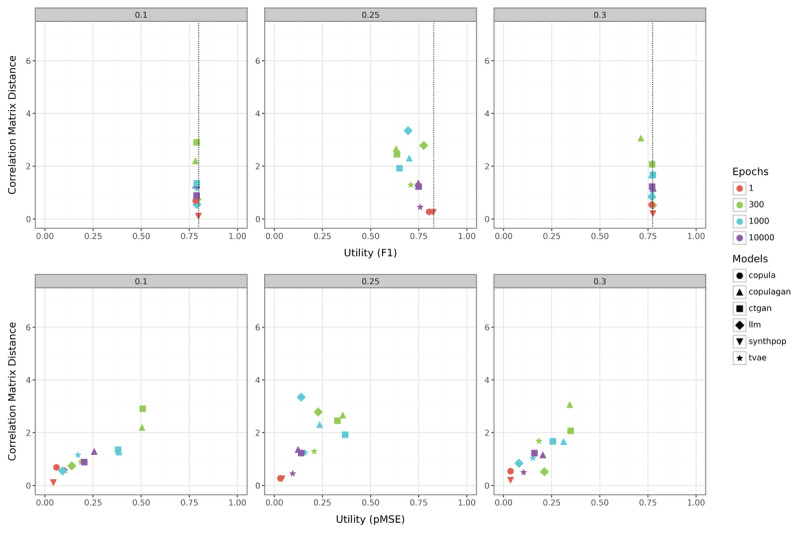
Comparison of the correlation matrix distance and utility metrics (F_1_-score in the top row; pMSE in the bottom row) for the simulated dataset with a sample size of 5000. ctgan: Conditional Tabular Generative Adversarial Network; LLM: large language model; pMSE: Propensity Score Mean-Squared Error; synthpop: Synthetic Populations in R; tvae: Tabular Variational Autoencoder.

Figure 5 illustrates the results obtained on the simulated dataset containing 5000 samples. It is evident that the increased dataset size improves the performance across all cases. Correlation matrix differences are smaller, and in the 0.3 case, almost all methods achieve similarly high levels of performance in terms of RF utility. Notably, the 0.25 case differs significantly from the other two cases, although its results are not substantially different from those observed with the 500-sample dataset. The most notable change is that copulagan and synthpop now emerge more clearly as the leading methods, whereas previously, tvae with high epochs had delivered comparable results. Overall, while deep learning methods benefit from the larger dataset, they still require a high number of epochs to perform well and do not yet match the performance levels of statistical methods.

**Figure 6:**
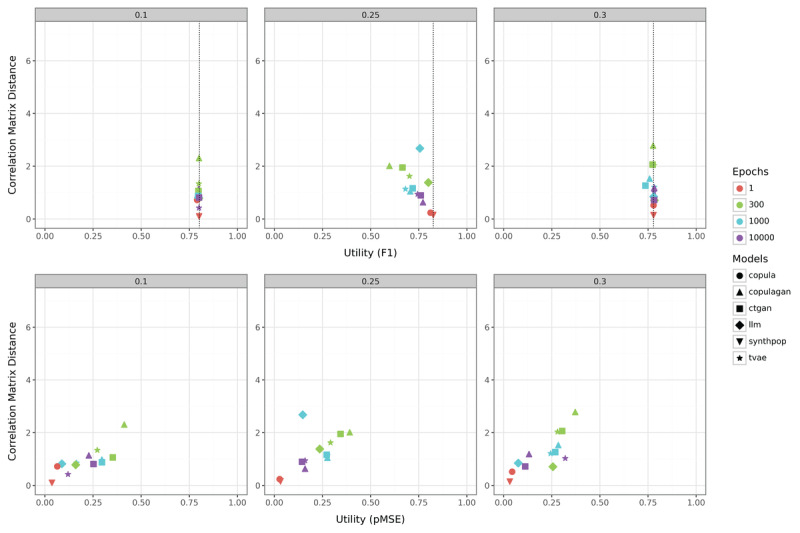
Comparison of the correlation matrix distance and utility metrics (F_1_-score in the top row; pMSE in the bottom row) for the simulated dataset with a sample size of 10,000. ctgan: Conditional Tabular Generative Adversarial Network; LLM: large language model; pMSE: Propensity Score Mean-Squared Error; synthpop: Synthetic Populations in R; tvae: Tabular Variational Autoencoder.

In the results of the simulation dataset comprising 10,000 samples, illustrated in Figure 6, the correlation matrix differences decrease slightly further. In addition, the performance of most deep learning methods improves in terms of RF utility and pMSE values when trained with 300 and 1000 epochs. Increasing the number of training epochs enhances the performance of deep learning methods more compared with 5000 samples but less compared to 500 samples. Otherwise, the results closely resemble those obtained with the 5000-sample dataset. This suggests that using a larger dataset for synthesis does not yield significant benefits unless the goal is to use deep learning methods with a limited number of epochs. However, the overall results indicate that such methods are generally not advantageous for datasets with a structure similar to that of our simulation study.

#### Real-World Data

**Figure 7:**
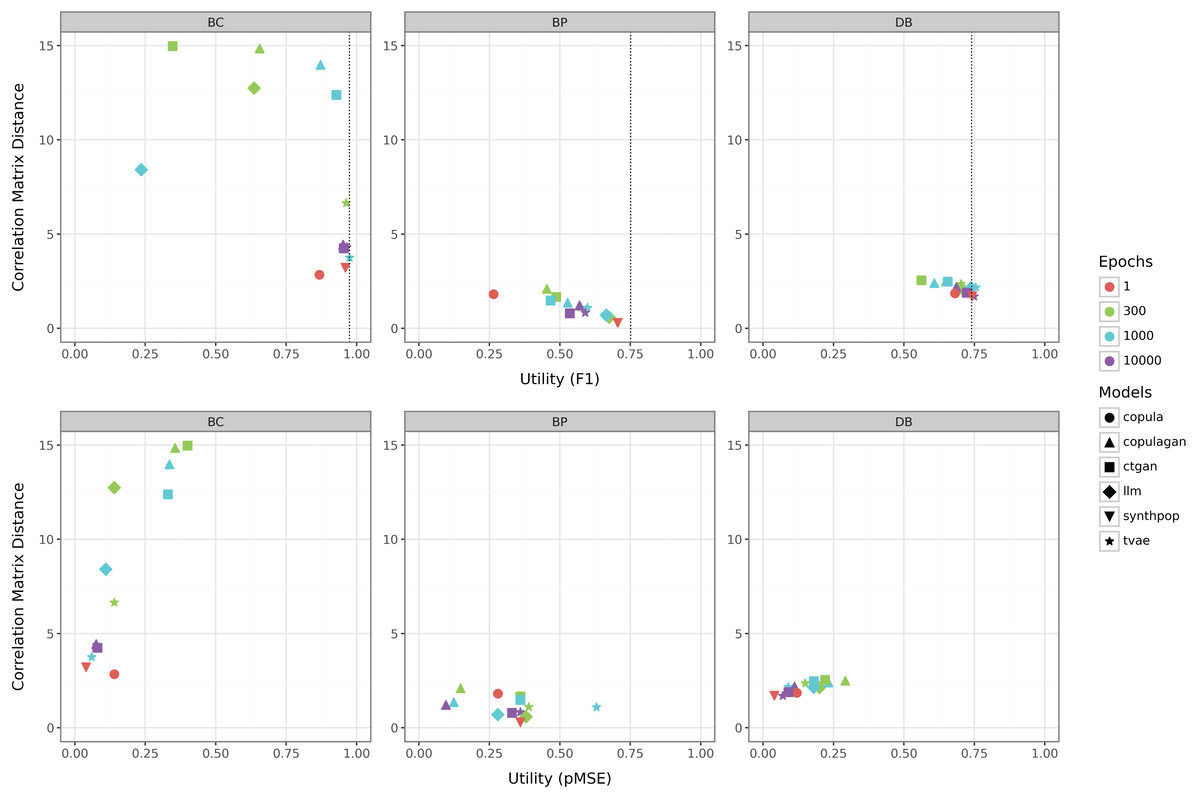
Comparison of the correlation matrix distance and utility metrics (F_1_-score in the top row; pMSE in the bottom row) for real-world datasets. BC: Breast Cancer Dataset; BP: Body Performance Dataset; ctgan: Conditional Tabular Generative Adversarial Network; DB: Diabetes Dataset; LLM: large language model; pMSE: Propensity Score Mean-Squared Error; synthpop: Synthetic Populations in R; tvae: Tabular Variational Autoencoder.

Due to the larger number of columns and a broader variety of data types in these datasets, the outcomes naturally exhibit some differences (Figure 7). Regarding the impact of dataset size, the results align closely with those observed in the simulated data for key trends. Specifically, smaller datasets exhibit significantly greater variability across all metrics. For the BC dataset, the copula method captures correlations most effectively, whereas synthpop achieves the best results in terms of RF utility and pMSE. BC is also the dataset where increasing the number of epochs benefits deep learning methods the most. This observation is consistent with findings from the simulated data, despite the real datasets featuring a considerably higher number of columns.

On the BP dataset, an initial observation reveals that copulagan achieves unexpectedly favorable pMSE values. This outcome becomes more comprehensible upon examining the dataset’s structure. While BP officially comprises 2 categorical variables (gender and class), it also includes sit-up counts, which is an integer variable that pose statistical modeling challenges. Estimating marginals using diverse distributions, such as the Beta distribution, as a preprocessing step for GANs, proves advantageous in this scenario, especially given the ample data available for these estimations. However, this does not translate into superior RF utility. The association between target and features is not adequately captured by copulagan, resulting in poor RF utility scores. In contrast, synthpop demonstrates the best RF utility and correlation matrix difference performance, although it struggles with achieving competitive pMSE due to the complexity of modeling integer variables. Copula, on the other hand, fails entirely to learn meaningful target-feature associations, yielding extremely low RF utility.

The DB dataset presents the fewest challenges to the methods overall, primarily due to the limited number of continuous variables it contains. All methods perform relatively similarly, reflecting the dataset’s inherent simplicity. Compared to the corresponding simulated dataset, one notable difference is that even methods with fewer epochs achieve relatively good performance. Otherwise, the insights gained from the 0.3 case simulation with 500 samples are largely transferable to this real-world scenario. Among the methods tested, synthpop and tvae demonstrate the best performance across all metrics, with synthpop again emerging as the most effective.

### Detailed Analysis of a Notable Result

We focus on the two least effective methods in terms of correlation matrix difference (ctgan with 300 epochs and LLM with 1000 epochs) and the best-performing method across all metrics (synthpop) on the 0.25 case of the simulated data consisting of 10,000 samples.

**Figure 8:**
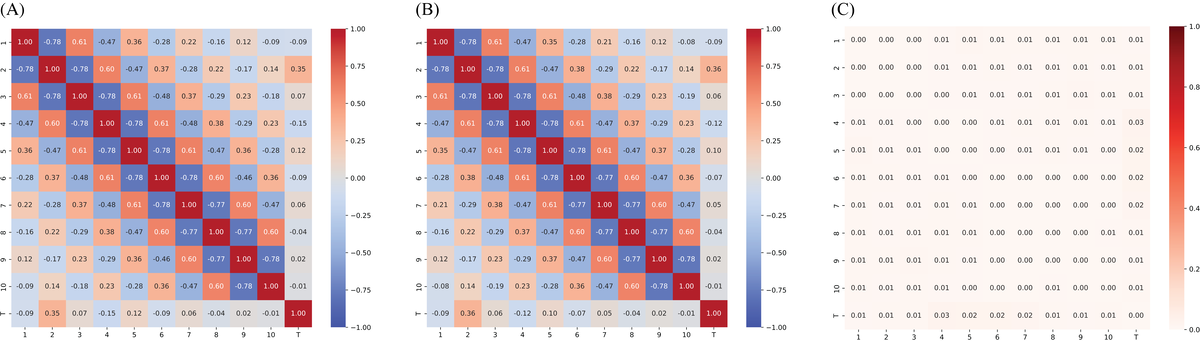
Correlation matrix of original simulated data (A), the mean correlation matrix of synthetic data (B), and the difference between (A) and (B) for synthpop with alternating correlation decay of 0.25 and sample size 10,000 (C). synthpop: Synthetic Populations in R

**Figure 9:**
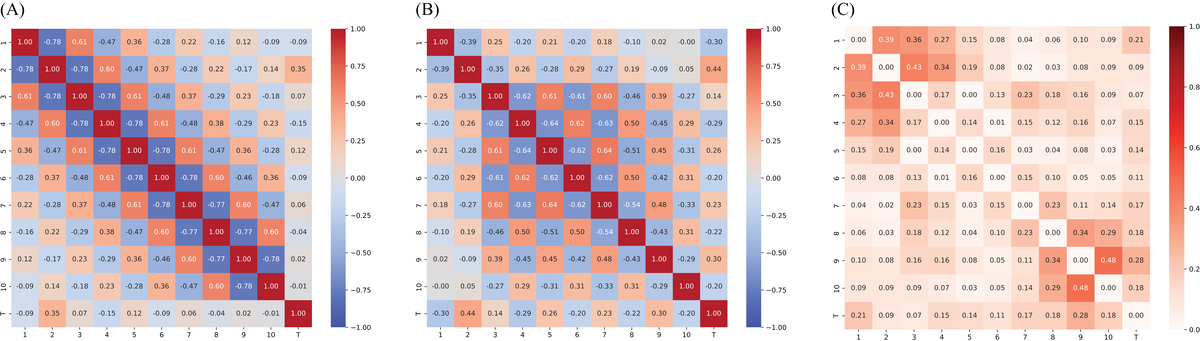
Correlation matrix of original simulated data (A), mean correlation matrix of synthetic data (B) and difference between (A) and (B) for ctgan with alternating correlation decay of 0.25, sample size 10,000, and 300 epochs (C). ctgan: Conditional Tabular Generative Adversarial Network.

**Figure 10:**
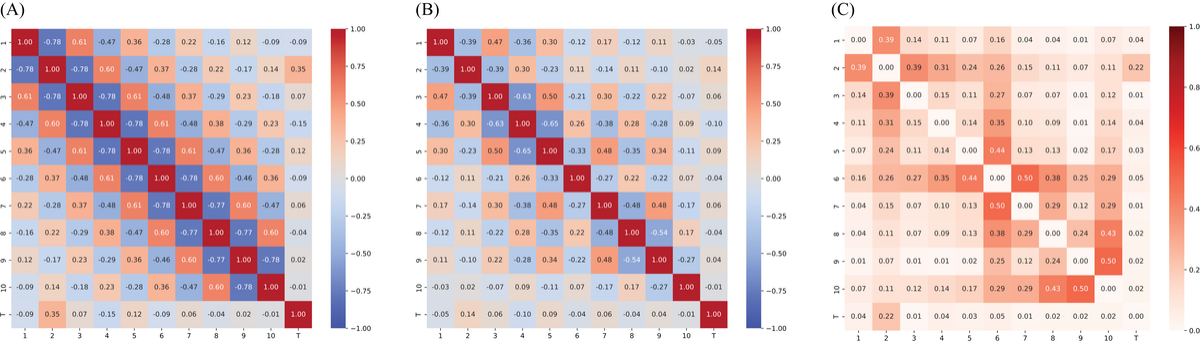
Correlation matrix of original simulated data (A), mean correlation matrix of synthetic data (B) and difference between (A) and (B) for LLMs with an alternating correlation decay of 0.25, sample size 10,000 and 1000 epochs (C). LLM: large language model.

**Figure 11:**
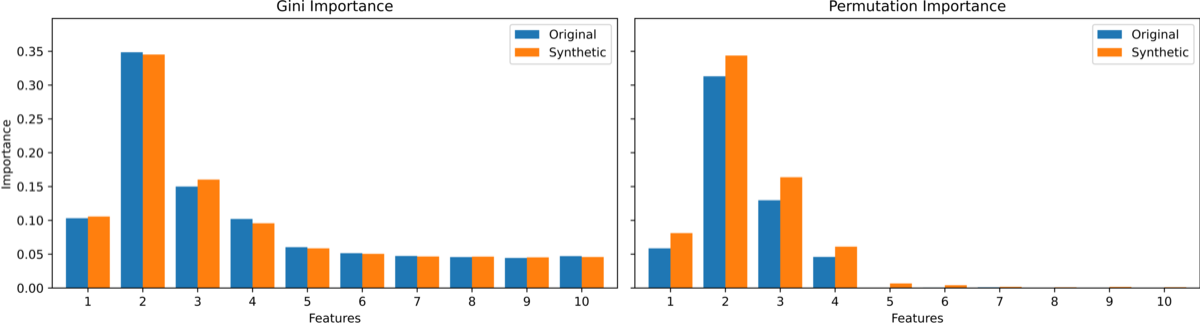
VIMP scores for original versus synthetic data generated using synthpop with an alternating correlation decay of 0.25 and a sample size of 10,000. Gini Importance (left) and Permutation Importance (right). synthpop: Synthetic Populations in R. VIMP: Variable Importance.

**Figure 12:**
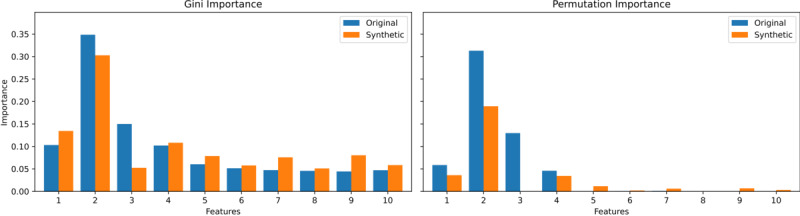
VIMP scores for original versus synthetic data generated using ctgan with an alternating correlation decay of 0.25, a sample size of 10,000, and 300 epochs. Gini Importance (left) and Permutation Importance (right). ctgan: Conditional Tabular Generative Adversarial Network; VIMP: Variable Importance.

**Figure 13:**
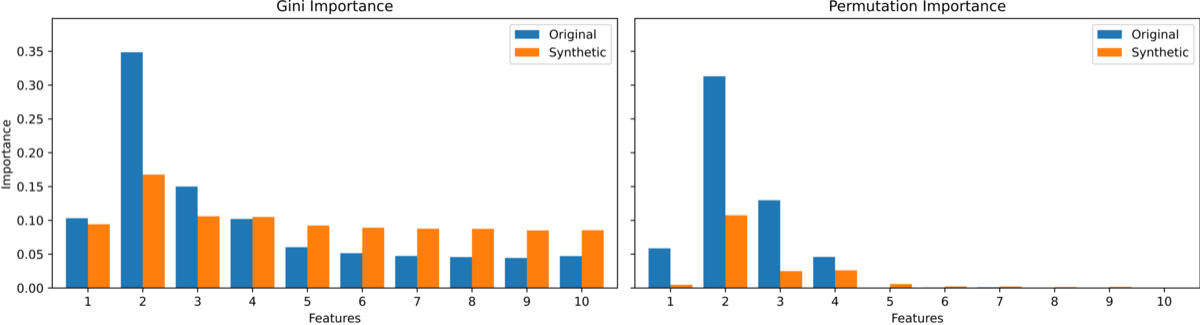
VIMP scores for original versus synthetic data generated using an LLM with an alternating correlation decay of 0.25, a sample size of 10,000, and 1000 epochs. Gini Importance (left) and Permutation Importance (right). LLM: large language model. VIMP: Variable Importance.

Figures 8-10 display the original correlations, those of the synthetic data, and the resulting correlation matrix differences for synthpop, ctgan, and LLM, respectively. While synthpop generates near-perfect synthetic data, both ctgan and LLM struggle, particularly with high absolute feature-feature correlations, which are often underestimated. In the case of LLM, this issue also extends to feature-target correlations, while ctgan exhibits feature-target correlations that exceed those in the original data. Overall, the underestimation of correlations is more pronounced in LLM than the mixed under- and overestimation seen in ctgan, which explains the larger correlation matrix differences observed in LLM. However, since the relative correlation ratios in LLM more closely resemble those in the original dataset, it performs better than ctgan in terms of RF utility and pMSE. Figure 11-13 display the VIMP scores (Gini and permutation importance) for synthpop, ctgan, and LLM, respectively. Synthpop shows near-identical results to the original data. The Gini importance for ctgan is promising, but the permutation importance reveals that feature 3 becomes entirely irrelevant. Features 7 and 9, due to their higher correlations with the target, are now relevant. For the LLM, feature 1 becomes nearly irrelevant. However, since feature 3 holds greater significance for the target variable, and no other irrelevant features exhibit substantial permutation importance, this does not detrimentally impact the RF utility or pMSE as severely as observed with the ctgan model. Overall, we conclude that large discrepancies in correlations harm utility only when the ratios between target and feature correlations shift significantly.

## Discussion

### Principal Findings

The central finding of our simulation study, which is largely transferable to real-world datasets, is that statistical methods such as copula and synthpop consistently outperform deep learning-based approaches across varying sample sizes and correlation complexities. Notably, synthpop emerged as the most effective method. These techniques demonstrate robust performance with minimal reliance on dataset size or extensive training, highlighting their reliability in preserving statistical properties and utility. However, our analysis of real-world datasets revealed that the copula method struggles when handling integer variables and increasing sample sizes does not mitigate this limitation.

In contrast, deep learning methods yield mixed results. While they benefit from larger datasets and extended training epochs, their performance often falls short of statistical methods, especially when trained with fewer epochs or on smaller datasets. These models struggle to capture the correlation structures, leading to higher pMSE values and diminished utility for downstream tasks. This suggests that deep learning models require careful tuning, including sufficient data and training time, to match the performance of statistical approaches. While the potential for deep learning models to handle datasets with diverse types is promising, the results presented here do not provide sufficient evidence to confirm this advantage over statistical methods. In addition, high performance observed for some deep learning-based approaches may be influenced by overfitting rather than genuine generalization.

The results obtained using the LLM method are somewhat disappointing. Despite a large sample size (≥10,000), this approach does not match the performance of synthpop. While the results are generally acceptable, they highlight that the sheer number of parameters in LLM models is not a decisive factor. Instead, methods specifically designed to directly replicate statistical properties and correlations are often more efficient and effective for tabular data. The probabilistic modeling of LLMs via next-token prediction reaches limitations, particularly when it comes to accurately replicating numerical dependencies. Although the attention mechanism offers promising potential, it does not directly address the preservation of distributions and correlations that are crucial for tabular data. In addition, the significantly longer runtime (hours instead of seconds or minutes), even with 2 high-performance NVIDIA H100 Graphics Processing Units, makes the use of the LLM method difficult to justify for our datasets. However, in cases where tabular data contains many features (more than 30), such as high-dimensional datasets, the runtime of synthpop (which runs on CPU) can become prohibitive when using classification and regression trees. In these cases, the runtime of LLMs may be comparable or even shorter, particularly as the number of rows increases.

Our detailed analysis of correlation matrix differences, VIMP scores, and utility uncovers one central mechanism that leads to either good or poor model performance. We find that a model’s utility is primarily influenced by the preservation of relative correlations between features and the target variable, rather than by large correlation matrix differences themselves. Although LLM exhibits greater correlation matrix differences after 1000 epochs compared to ctgan after 300 epochs, this does not result in worse utility. This is because LLM better preserves the relative correlations, particularly those between the features and the target, which leads to improved RF utility and pMSE. In contrast, while ctgan shows good Gini importance values, its less accurate representation of the correlation value ratios has a greater negative impact on utility. Overall, our findings demonstrate that it is not the absolute magnitude of correlation matrix differences, but the relative correlations between features and the target variable that are critical for model utility.

Our results confirm those found in the literature [36,37] but extend them by incorporating LLMs for the first time and using a simulation approach to assess the impact of various correlation structures on the outcomes. Statistical techniques, such as copula and synthpop, are widely recommended for medical datasets with characteristics similar to those in this study. However, our analysis of the BP dataset highlights the potential usefulness of deep learning methods, particularly when handling multiple variables of diverse data types. In these scenarios, deep learning approaches are anticipated to be able to outperform both synthpop and copula-based methods.

### Limitations

A key limitation of this study is that our simulation focused primarily on pairwise correlations. This decision was intentional, as we aimed to restrict our exploration to a small set of scenarios to maintain manageable complexity and derive initial insights. While many of our findings translated well to real-world data, the BP dataset highlighted an important challenge: when dealing with more complex scenarios involving a larger number of variables, diverse data types, and intricate interaction patterns, such as those commonly found in omics or high-dimensional datasets, it becomes essential to design advanced simulation studies that better capture these complexities [38]. In such cases, conventional approaches like Cholesky decomposition or even copula-based methods may no longer suffice [39].

Another limitation of our work is the exclusion of more recent and potentially transformative methods, such as diffusion models [40]. These models have demonstrated exceptional performance in generating high-quality synthetic data, particularly for images, and their application to tabular data represents a promising direction for future research. Moreover, we did not extensively evaluate how our chosen methods perform under scenarios involving temporal or longitudinal data, multimodal datasets, or extreme imbalance in class distributions, challenges that are increasingly relevant in modern data science applications. Addressing these aspects would provide a more comprehensive understanding of the strengths and limitations of SDG methods in diverse contexts.

Further, privacy considerations were not evaluated as part of the synthetic data generation process. While the generative models aimed to preserve data utility and structural similarity, privacy risks such as data leakage or membership inference attacks were not assessed due to our focus in the relationships between correlation structure and utility under different scenarios.

Finally, in synthetic data generation, it is critical to account for biases. If the original data contains biases, the synthetic data is likely to mirror these, potentially leading to discriminatory health care outcomes, particularly for marginalized or underrepresented groups. To mitigate such risks, bias detection and adjustment techniques, such as reweighting, oversampling, and fairness constraints, should be integrated into the data generation process. Beyond bias, ethical concerns also include privacy, informed consent, and accountability. For instance, transparency in the data generation process and clear, informed consent from data contributors are essential for maintaining ethical standards. Regular audits of the synthetic data and associated models are necessary to identify and correct emerging biases and privacy breaching risks.

### Conclusions

Statistical methods, particularly synthpop, consistently outperform deep learning–based approaches in preserving statistical properties and utility across diverse datasets, establishing their robustness and reliability. Copula methods show promise but struggle with integer variables, limiting their application in real-world scenarios. Deep learning methods, while resource-intensive and sensitive to hyperparameters, may outperform statistical approaches in handling highly complex datasets with mixed variable types when sufficient training samples and computational resources are available. LLMs, despite their theoretical potential, demonstrated suboptimal performance and high computational costs for the datasets analyzed in this study. Overall, these findings underscore the dominance of statistical methods for synthetic data generation for tabular data, while highlighting the niche potential of deep learning approaches for highly complex datasets, provided adequate resources and tuning.

## Data Availability

Data are deposited in publicly available repositories (where available and appropriate).

## Appendix

Multimedia Appendix 1 F1 Scores for RF, XGBoost, and GATE Across All Datasets Synthesized Using SDG Methods with Varying Configurations (Epochs and Batch Sizes).

## Abbreviations

BC: Breast Cancer Dataset
BP: Body Performance Dataset
ctgan: Conditional Tabular Generative Adversarial Network
DB: Diabetes dataset
DistilGPT-2: distilled Generative Pretrained Transformer-2
GAN: Generative Adversarial Network
LLM: large language model
ML: machine learning
pMSE: Propensity Score Mean-Squared Error
RF: Random Forest
SDG: Synthetic Data Generation
SDV: Synthetic Data Vault
SynthPop: Synthetic Populations in R
TVAE: Tabular Variational Autoencoder
VIMP: Variable Importance
